# Ergonomic assessment of the posture of surgeons performing endoscopic transurethral resections in urology

**DOI:** 10.1186/1745-6673-4-26

**Published:** 2009-10-19

**Authors:** Alwin Luttmann, Matthias Jäger, Jürgen Sökeland

**Affiliations:** 1 IfADo - Leibniz Research Centre for Working Environment and Human Factors, Ardeystraße 67, 44139 Dortmund, Germany; 2 Urologische Klinik, Städtische Kliniken Dortmund (formerly), Münsterstraße 240, 44145 Dortmund, Germany

## Abstract

**Background:**

During transurethral endoscopic prostate and bladder operations the influence of an ergonomic redesign of the arrangement of the operation equipment - including the introduction of a video-assisted resection method ('monitor endoscopy') instead of directly viewing onto the operation area via the endoscope ('direct endoscopy') - was studied with respect to the postures of the surgeons.

**Methods:**

Postures were analysed on the basis of video recordings of the surgeons performed in the operation theatre during live operations and subsequent visual posture estimation executed by an observer. In particular, head, trunk and arm positions were assigned to posture categories according to a newly developed posture classification schema. 10 urological operations with direct endoscopy and 9 with monitor endoscopy were included.

**Results:**

Application of direct endoscopy coincides with distinct lateral and sagittal trunk and head inclinations, trunk torsion and strong forearm and upper arm elevations of the surgeons whereas operations with monitor endoscopy were performed with an almost upright head and trunk and hanging arms. The disadvantageous postures observed during direct endoscopy are mainly caused by the necessity to hold the endoscope continuously in close contact with the eye.

**Conclusion:**

From an ergonomic point of view, application of the video-assisted resection method should be preferred in transurethral endoscopic operations in order to prevent awkward postures of the surgeons and to limit muscular strain and fatigue. Furthermore, the application of the monitor method enables the use of a chair equipped with back support and armrests and benefits the reduction of postural stress.

## Background

### Historical development of endoscopic operation methods in urology

The application of endoscopic operation methods has a long tradition especially in urology. As early as 1879 optical endoscopy began when the urologist Maximilian Nitze demonstrated the first rod-shaped cystoscope equipped with an optical lens system and an electrical light source (for historical review of cystoscopy see [1,2]). In the following decades the instruments were improved by introducing light transmitting glass fibres for the illumination and so-called 'air-lens or rod-lens systems' for the visual inspection of the operation area.

Since the first application of such a cystoscope in the last decades of the 19^th ^century until the eighties of the 20^th ^century, i.e. for about 100 years, only "direct endoscopy" was applied in urology. Using this method, one of the surgeon's eyes looks through the optical lens system directly into the body and the eye is permanently in contact with the ocular of the endoscope. In consequence, the head of the surgeon has to follow all movements of the instrument. From an ergonomic point of view, direct endoscopy has to be assessed as disadvantageous, since awkward postures are inevitable, which are at least partially caused by the construction of the instruments.

**Figure 1:**
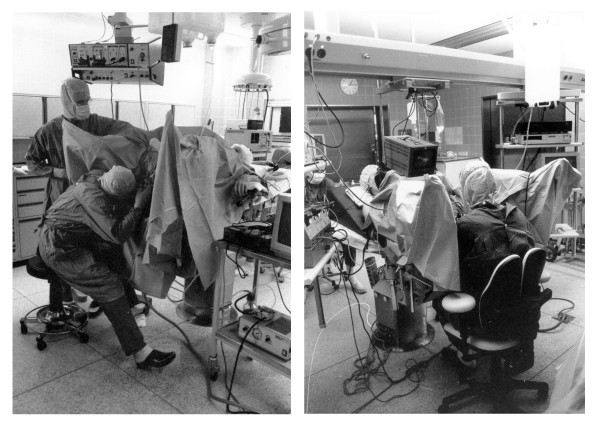
**Photos of the operating theatre before and after redesign**. Photos illustrating the arrangement of the operating devices and typical postures of the surgeon before (left) and after (right) ergonomic redesign of the workplace including the introduction of a video system for the control of the operation area.

For transurethral applications in urology 'resectoscopes' are used consisting of the endoscope and a wire loop which is mounted together with the endoscope in the same shaft. For the dissection of tissue from the bladder or prostate the wire loop is - under control of a foot-switch - charged with a high-frequency current and moved through the tissue. The resectoscope is held with one hand and the other is used to manipulate the wire loop. The application of this method results in a 'close coupling' between the head, the hands, and the endoscope. Since in direct endoscope the eye has to be permanently in contact with the instrument, the surgeon has to steeply incline the upper body for long periods of the operation, in particular during the resection of tissue from the ventral part of the prostate or bladder. A typical posture of the surgeon with inclined head and trunk while the eye is in close contact with the resectoscope is shown in the left-hand photo of figure 1. The performance of transurethral resections requires high motoric dexterity on the part of the surgeons, since even in disadvantageous postures of the upper body, fine movements of the hands and fingers have to be executed.

### Modern endoscopic instrumentation using video technique

With respect to postural load of the surgeons, improvement is attained, if instead of the direct-view endoscopy the "monitor endoscopy" is applied. In this method the visual inspection of the operation area is performed via a video camera mounted on the top of the endoscope and a monitor. Such video devices are available since the beginning of the eighties of the last century and meanwhile monitor-displayed endoscopy can be assessed to be the golden standard for transurethral resections [3]. The ergonomic advantage of this method is that the complication of the close coupling between the eye and the endoscope is solved and the surgeon's trunk and head can remain in an almost upright posture during the operations (see photo in the right-hand part of figure 1).

In the first years after their introduction, the video systems were used only hesitantly in urology [3]. The delayed application of the monitor method may at that time be caused by an insufficient ergonomic arrangement of the technical devices. In particular, the monitor was often placed on a movable trolley at the side of the operating table or was hanging on the ceiling outside the central visual field of the surgeon. So the visual inspection of the monitor requires disadvantageous postures with long-term twisting of the neck and trunk and the arrangement results in deficiencies in the perception-action compatibility [4].

### Ergonomic redesign of the urological operation theatre

The study on hand was performed before and after a comprehensive redesign of the operation theatre (see right-hand part of figure 1) and was aimed to evaluate the rearrangement with respect to the surgeons' postures. Redesign was performed for several reasons: One reason was to support the routine application of the video system and to reduce the load on the musculoskeletal system. A fast and easy control of all visual readouts including the video monitor should be possible without turning or twisting the trunk and head. Furthermore, a flexible arrangement of the tools and devices needed for transurethral operation was aspired. The placement of the equipment should allow to have free access to the patient when performing various operations. At least, the arrangement including the operating chair should be adaptable to the individual anthropometry of the surgeon and the patient. The demands were fulfilled by applying a horizontal bar fixed to the ceiling of the operation theatre; all devices including the monitor were mounted on racks hanging on the bar. The racks can slide horizontally on the bar and can be arranged according to the actual requirements of a special operation. The monitor position can also be changed in the horizontal as well as in the vertical direction. In the vertical direction, positioning between the hand and eye level is strived for, according to the anthropometry of the surgeon. Nevertheless positioning above eye level is often inevitable, since a certain minimum distance of the monitor above the patient's chest has to be maintained. Horizontal fixation of the monitor in the mid-sagittal plane of the surgeon allows the control of the operation area without turning or twisting the head or trunk and benefits the hand-eye coordination [5]. A special chair equipped with adjustable back- and armrests was applied in order to reduce postural stress further (for details, see [6]).

### Posture recording

Even if the advantage of the monitor method seems to be obvious with respect to ergonomic work design, a quantitative analysis of the postures which surgeons adopted during the performance of transurethral resections is missing. Therefore, the aim of this study was to determine the postures of urologists during the execution of transurethral operations and, in particular, to compare the postures during operations with direct and monitor endoscopy.

For the recording of posture, sophisticated methods were developed, consisting e.g. of goniometers and inclinometers to measure the inclination and flexion of body segments (e.g. [7]) or optoelectronic devices using reflective markers or light-emitting diodes to track relevant body locations in combination with a set of video- or infrared cameras. Such methods need high effort in applying the posture-recording technique and can hardly be adapted to the specific demands of an application in the operation theatre during surgical treatments (e.g. to ensure sterility and to avoid any restrictions of surgical actions). For such reasons, continuous recording of posture data during real surgical work was executed rarely. However, the possibility of such measurements was demonstrated in a pilot study with optoelectronic posture measurements during laparoscopic cholecystectomy under live conditions [8]. Similar measurements using optoelectronic devices were performed during operation-simulation experiments [9]. In another approach, posture was determined for defined sections of live operations on the basis of sequences of photos [10] or characteristic long-lasting static postures were selected using video recordings and assessed using a computerised man model [11]. The method used in the study on hand represents an extended version of a posture estimation procedure applied by Berguer et al. [9]. It is based on video recordings of the surgeons taped during live surgical work in an operating room and a visual off-line estimation of the surgeons' postures performed by an observer. This posture-estimation method was favoured in spite of its limited accuracy, since the surgeons activity was not influenced by the measuring technique and the procedure was easy to apply under live conditions in the operating theatre.

Posture documentations were used to evaluate the postural stress of the surgeons during live surgical work in urology before and after redesign of the operation theatre in particular regarding the introduction of modern endoscopic instrumentation. It is concluded, that the use of a video system results in a clear reduction of awkward positions of the upper body segments and is therefore recommended for transurethral endoscopic operations.

## Methods

### Study protocol and subjects

The study was performed before and after an ergonomic redesign of the arrangement of the operational equipment. Before redesign direct endoscopy was applied only. More than one year after redesign, investigations regarding monitor endocopy were executed, when the surgeons were getting well accustomed to the new arrangement and almost exclusively used the video system.

In total, 5 surgeons (male; age between 34 and 61 years, mean 45 years; body height between 172 and 190 cm, mean 182 cm) participated in the study. Analyses were carried out for 10 operations with direct endoscopy (7 treatments of prostate adenoma; 3 resections of bladder tumours) and for 9 operations with monitor endoscopy (5 treatments of prostate adenoma; 4 resections of bladder tumours). The analysed operation periods lasted between 18.1 and 79.4 min (mean 41.1 min) for direct endoscopy and between 12.8 and 75.6 min (mean 37.8 min) for monitor endoscopy.

### Posture recording and categorisation

**Figure 2:**
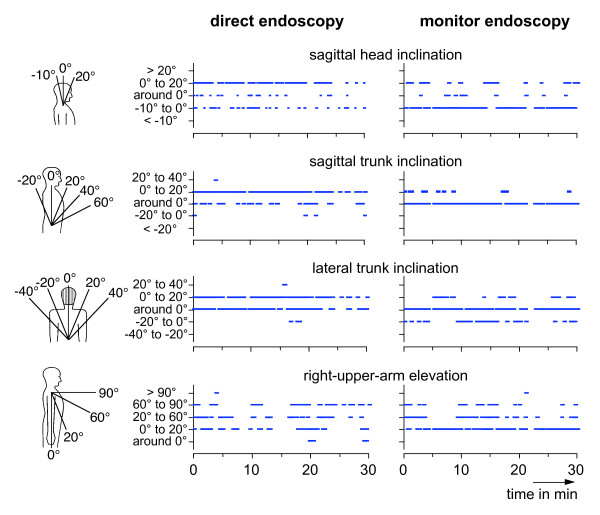
**Time course of posture indicators**. Examples of the time courses of various posture indicators for the first 30 min of an operation performed with direct endoscopy (left) and monitor endoscopy (right). The used posture categorisation is illustrated in the body-contour drawings.

Video recordings of the surgeons' postures were performed in the operation theatre continuously throughout the operations. A video camera was mounted in a height of about 2 1/2 meters on a tripod standing at the right-hand side behind the surgeon. The camera looked from above on the surgeon and was focused on the upper part of the body and the right arm-hand region. The view on the left arm and hand was often restricted. The analysis of the video recordings was executed subsequently in the laboratory by visual inspection of the videos and applying an encoding system for the description of the consecutive postures the surgeon has adopted in the course of the operation. Whenever the surgeon has changed his position, the analysing person had to estimate the current position of the surgeon and to create a numerical code. In this code, the angular positions of various body segments were described using a specific classification procedure. This classification system is based on a method which was originally developed for the posture evaluation of manual handling tasks [12]; it was modified and adapted to the actual problem. An overview of the classification system is provided in table S1, additional file 1. It is based on a "16-digit posture code" used to describe the posture of the trunk (5 digits), head (4 digits) and the right shoulder and arm (6 digits). Additionally the posture of the lower body (1 digit) and the line of vision (1 digit) were documented. The task of the analysing person was to assign the actual posture of the body segments mentioned in table S1, additional file 1 into given categories. With regard to sagittal trunk inclination (digit 1) the actual position of the trunk is classified in steps of 20° between 60° forward and 20° backward, i.e. the categories '>60°', '40° to 60°', '20° to 40°', '0° to 20°', 'around 0°', '-20° to 0°' and '<-20°' are provided. For the category 'around 0°' a span between about 5° forward and backward is assumed. Accordingly, 7 expressions were used to quantify the sagittal trunk inclination (see column 5 in table S1, additional file 1). Similarly, the trunk posture was categorised in 20°-steps with respect to the lateral inclination and torsion. (For clarification, see also the body-contour drawings in figure 2 illustrating some posture categorisations exemplarily.) Adoption of a hollow back and the use of the back support of the chair ('trunk support') were categorised using two expressions ('yes' or 'no'). The postures of the head were codified analogously using the digits 6 to 9; for the sagittal and lateral head inclination also 20°-steps were chosen with the exception of the backward inclination where categories of '0° to -10°' and '< -10°' are provided. The positions of the right shoulder and arm were classified using the digits 10 to 15. The posture of the lower body was described in digit 16 with only 2 expressions ('sitting' or 'standing'), and digit 17 was applied to discriminate between different lines of vision and was used in operations with monitor endoscopy, only. The application of the encoding system to the 19 operations considered in this study reveals a mean number of encoded segment positions of about 7,500 per operation ranging between 2,500 and 14,700.

The posture categorisation is based on the subjective estimation of an analysing person and not on measurements using technical sensors. For this reason, a restrained application of the method is indispensable and a critical appraisal is provided in the discussion section. In the conception of the study, some measures are undertaken in order to limit the risk of errors; e.g. only one person has performed the posture encoding for all operations in order to prevent differences in the posture categorisation caused by different interpretations of various analysing persons. Furthermore, the analysing person was intensively trained before executing the posture encoding and the reproducibility of her posture rating was checked.

## Results

### Example for the time courses of upper-body segments' positions

The encoding procedure results in a series of consecutive code numbers representing the time course of the posture of relevant body segments. In figure 2 examples of the resulting time sequences are shown graphically for the head, trunk and upper arm postures during the first 30 minutes of two operations, one with direct endoscopy (left-hand diagrams) and another with monitor endoscopy (right-hand diagrams). Additionally, a graphical illustration of the posture categorisation is shown at the left-hand side of the figure using body-contour drawings. In the upper trace the time courses of the head inclination in the sagittal plane are presented. Postures were assigned to only three categories with angles between -10° and 20°. The category '0° to 20°', indicating a slight forward inclination, was preferable quoted in case of direct endoscopy, whereas for monitor endoscopy backward inclinations between -10° and 0° were most frequently mentioned. In the two middle traces in figure 2 the time courses of the sagittal and lateral trunk inclinations are indicated. For direct endoscopy, the highest number of entries was found for sagittal as well as lateral inclinations in the class of '0° to 20°'. For monitor endoscopy, the class 'around 0°' was quoted most frequently for both directions. In the lowest trace of figure 2 the time course for the right-upper-arm elevation is presented. For both operation techniques high fluctuations of the elevation angle can be seen with a larger variation range for direct endoscopy than for monitor endoscopy.

### Summarising results regarding head, trunk and arm positions

**Figure 3:**
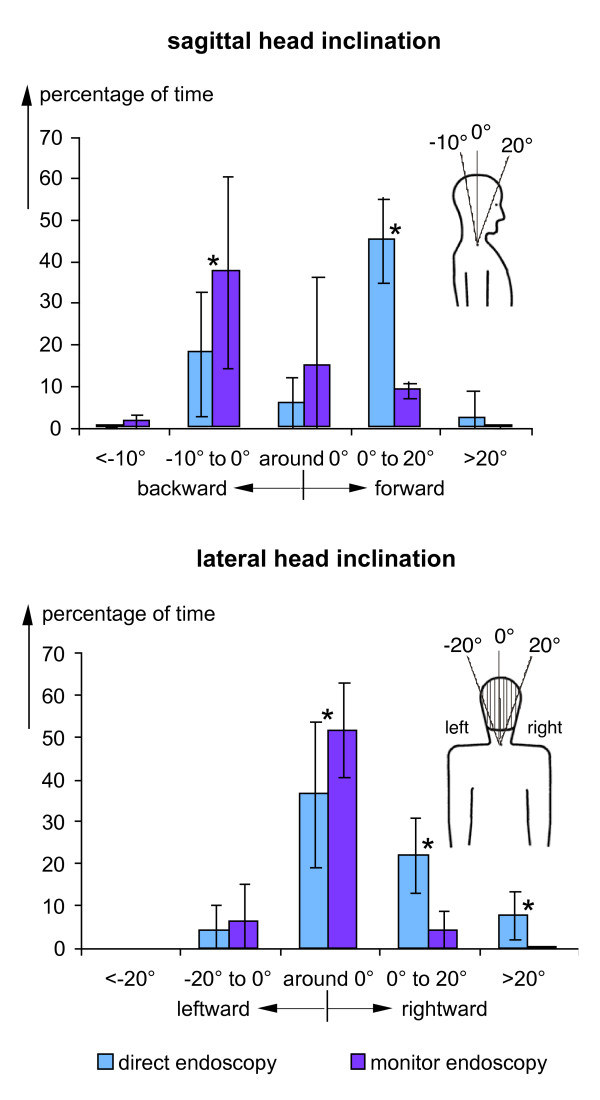
**Frequency distributions of head inclination**. Comparison of histograms of the fractions of time with head inclination in the sagittal (above) or lateral plane (below) for operations with direct and monitor endoscopy - mean and standard deviation (direct endoscopy: n = 10, monitor endoscopy: n = 9), * = significantly different (p < 0.05). Specifications of the head inclination angles are provided in the insets.

**Figure 4:**
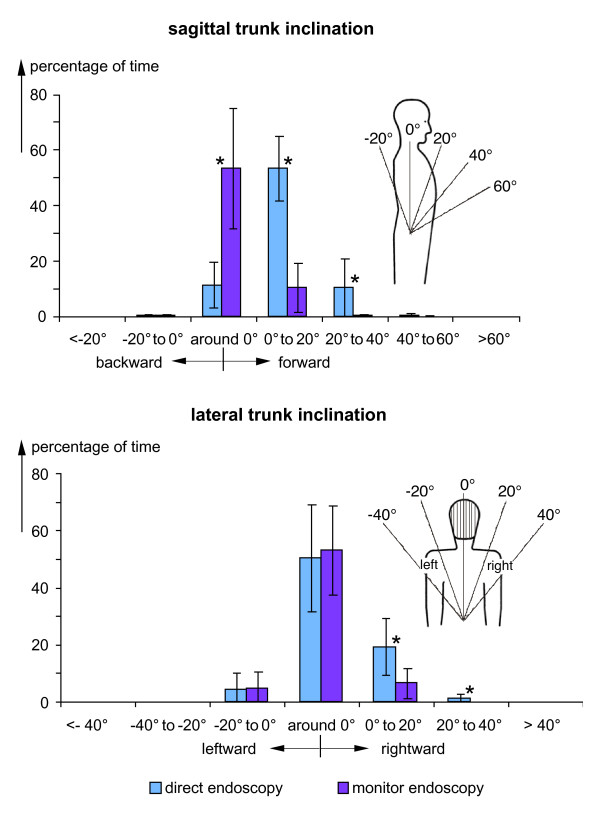
**Frequency distributions of trunk inclination**. Comparison of histograms of the fractions of time with trunk inclination in the sagittal (above) or lateral plane (below) for operations with direct and monitor endoscopy - mean and standard deviation (direct endoscopy: n = 10, monitor endoscopy: n = 9), * = significantly different (p < 0.05). Specifications of the trunk inclination angles are provided in the insets.

**Figure 5:**
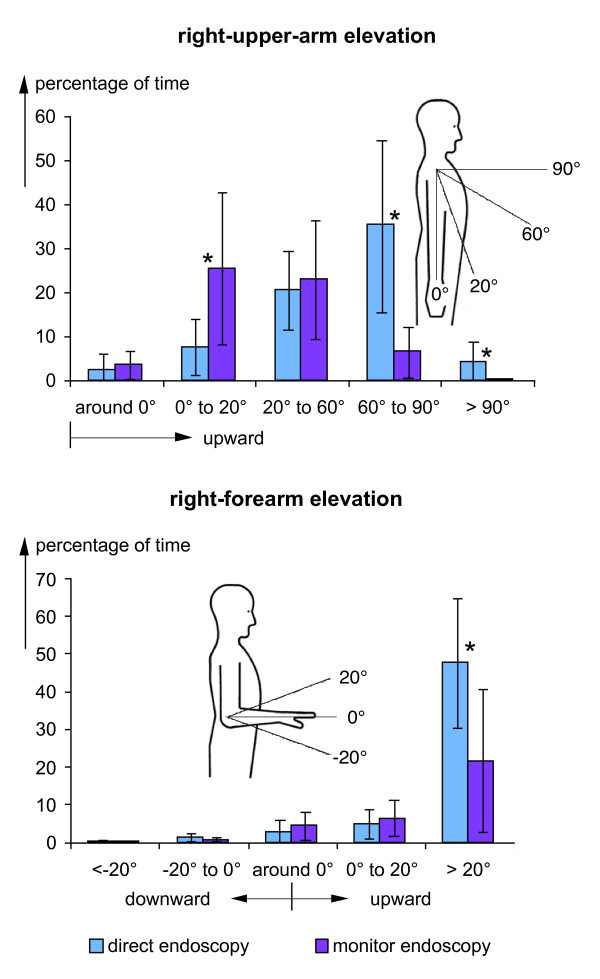
**Frequency distributions of right-arm elevation**. Comparison of histograms of the fractions of time with elevation of the right upper arm (above) or the right forearm (below) for operations with direct endoscopy and monitor endoscopy - mean and standard deviation (direct endoscopy: n = 10, monitor endoscopy: n = 9), * = significantly different (p < 0.05). Specifications of the arm elevation angles are provided in the insets.

The findings demonstrated exemplarily in figure 2 for single surgical treatments were summarised for all analysed operations. Results are presented in figures 3, 4 and 5 in the form of histograms for the fractions of time for the various categories of the head and trunk inclinations as well as of the elevations of the right upper arm and forearm. The fractions of time were determined for each operation as the total time spent in the specific posture categories and expressed as the percentage of the duration of the respective operation. The fractions of time were averaged over all operations and are presented in the figures 3, 4 and 5 together with the respective standard deviations and the result of the statistical comparison between both operation methods (t-test).

In figure 3 the histograms of the percentages of time spent in postures with head inclinations are shown for both, the sagittal (upper diagram) and lateral direction (lower diagram). For direct endoscopy, the maximum in the frequency distribution exists in the sagittal plane for forward inclinations of 0° to 20° and, for monitor endoscopy, in backward direction between -10° and 0°. For both operation techniques distinct skew frequency distributions were found; this implies that during direct endoscopy forward inclinations of between 0° and 20° occurred most often but were observed rarely above 20°, whereas during monitor endoscopy the highest frequency was found for backward inclinations between -10° and 0° and inclinations below -10° were avoided. For lateral head inclinations (lower diagram) the maximum in the frequency distribution is located in the class 'around 0' for both operation techniques. The distributions differ, however, in so far, as the distribution is almost symmetrical for monitor endoscopy, whereas for direct endoscopy inclinations were found more often in rightward than in leftward direction.

Histograms for the sagittal and lateral trunk inclinations are presented in figure 4. For the sagittal plane (upper diagram), differences regarding the location of the maximum were found: During direct endoscopy most situations coincide with slight forward inclinations in the category '0° to 20°' and for monitor endoscopy upright positions with an inclination angle 'around 0°' were preferably chosen. In the lateral plane (lower diagram), the distribution is almost symmetrical for monitor endoscopy whereas for direct endoscopy the persons adopted postures with rightward inclinations for a higher percentage of time. Besides the sagittal and lateral trunk inclination its torsion was evaluated (not shown in the figure). The percentage of time with trunk torsion amounted to about 10% for direct endoscopy and was clearly reduced to about 2% when the monitor method was applied.

In figure 5 the findings regarding the right-arm positions are summarised. Differences between the operation techniques were found in particular for the right-upper-arm elevation (upper diagram) where distinct skew frequency distributions were observed with a maximum in the class '60° to 90°' for direct endoscopy and of '0° to 20°' for monitor applications. Also the percentage of time with right-forearm elevations of more than 20° is clearly increased during direct endoscopy (lower diagram). Comparison of the postures for the different operation methods included the observation of elbow flexions (not shown in the diagram). In particular for extreme flexions of more than 90°, a reduction from about 50% to about 20% was found when the monitor method was applied instead of direct endoscopy.

As a part of the redesign of the equipment, a newly developed operating chair equipped with a back support and armrests was introduced. It was applied in the video-assisted operations, only. Evaluation of the video recordings reveals, that the back support was intensively used during monitor endoscopy for about 34% of the operation time whereas the right-hand armrest was used for only 3% of the time. The newly developed chair was not applied during the operations with direct endoscopy, since the back support and the armrests may hamper the free movement of the trunk and arms which is necessary to enable the permanent contact between endoscope, eye and hands.

In the figures 3, 4 and 5 the fractions of the operation time, i.e. the sum of all time periods with defined postures in relation to the total operation time, are provided as an indicator of the postural stress of the surgeons. Besides these fractions of time found by cumulating the duration of all time periods with specific postures during an operation, the mean length of the time periods the surgeons remained uninterruptedly in the various posture categories were determined for each operation. The findings were averaged over all operations with direct and monitor endoscopy, respectively. Results are presented in table S2, additional file 2, for the various head and trunk inclinations in the sagittal and lateral directions and for the elevations of the right upper arm and forearm. Additionally, the ranges for the means found for the single operations are presented in brackets. Comparison of the mean duration of the time periods in the various postures table S2, additional file 2, with the corresponding frequency distributions of the cumulated periods (figures 3, 4 and 5) yields a clear similarity between both measures of postural stress; in particular, the maximum values in the durations and the time fractions are to be found for the same postures.

## Discussion

### Criticism of the method

The posture description in this study is based on an easy-to-apply method of video recording and a subjective posture rating based on visual inspections of the screen images. The use of such a seemingly simple method needs critical assessment. The advantage of this procedure is based on the low technical demands and the fact that the work of the surgeons is not constrained in any way. A possible disadvantage originates from the limited accuracy of the posture description which depends, on the one hand, on the ability of the observer to distinguish between different postures and, on the other hand, on the step-width of the angles used in the posture categorisation procedure. When defining the classification steps a compromise has to be found between these two factors. A relatively high step-width of 20° was chosen in order to enable visual inspection. Such a difference of 20° between segment positions can clearly be differentiated, even if spatial movements of the body segments have to be analysed and only a two-dimensional reproduction on a screen is used. With respect to the precision of the rating it has to be considered that the evaluation is not focussed on the absolute inclination angles of the body segments but on the discrimination between the postures in the two workings situations with direct and monitor endoscopy. Therefore, the requirements regarding the resolution of the inclination angles and the need of correct angle values are limited and a step-width of 20° was assessed as being sufficient for the classification.

In order to benefit the reproducibility of the categorisation and to prevent inter-individual differences in the posture estimation the encoding procedure was executed by the same person for all operations. This person was intensively trained by multiple executions of analyses of the same video recordings and checking the reproducibility of the ratings of identical video sequences. The differences between the categorisation of identical segment positions amounted - if at all - to one classification step, only. Insofar experiences from previous application of a visual video-based posture rating [12] are confirmed. The relatively high reproducibility of such a subjective posture rating is to a high degree based on the off-line encoding of the video recordings which allows multiple inspections of the same activity section and a stepwise rating of the posture of the various body segments.

### Postural stress for different operation techniques

The differences in the postures found for the two operation methods can be explained mainly by the different handling of the resectoscope in direct and monitor endoscopy and the necessity for the eye to remain continuously in contact with the ocular of the instrument when applying direct endoscopy.

Distinct forward and rightward inclinations of the trunk and head as well as twisting of the trunk were found in direct endoscopy, since in this operation technique a close coupling of instrument, hands and eye is permanently needed and the trunk and head have to track the endoscope movements during the total operation time. Therefore, awkward postures cannot be avoided, particularly if tissue has to be removed at the ventral part of the prostate or bladder. During monitor endoscopy, a clear backward inclination of the head was observed for considerable percentages of time, since the monitor was positioned above eye level for a part of the surgeons. Positioning at a lower level was, however, possible only to a limited extent, since the bottom of the monitor has to stay away from the thorax of the patient in a certain minimum distance. In the sagittal plane, the trunk and head remained mostly in an upright position when a monitor system was used and sideward inclinations - frequently found during direct endoscopy - were avoided. The angles of upper-arm and forearm elevations were remarkably higher during direct endoscopy than during monitor use. In direct endoscopy, permanent arm elevation is required to hold the endoscope for prolonged periods in time in close contact with the eye, whereas the use of a video system allows to work with almost hanging upper arms.

In the figures 3, 4 and 5 the results of the statistical comparison of the fractions of time spent in the various posture categories during direct and monitor endoscopy are indicated. For all body segments and most of the relevant posture categories a significant reduction of the fraction of time spent in awkward postures was found for operations with monitor endoscopy. Thus, the ergonomic improvement of the redesign of the workplace including the introduction of the monitor endoscopy is substantiated.

Some of the standard deviations shown in the figures 3, 4 and 5 are relatively high and were matter of further analyses, in particular, regarding the influence of the body height of the surgeons. For this purpose the percentage of time in the various posture categories were determined separately for the surgeons with a body height below and above 180 cm (2 and 3 persons, respectively). Significant difference was found for the percentage of time with backward inclinations of the head during monitor endoscopy amounting to more than 60% for the smaller persons in comparison to about 20% for the taller ones. Further significant differences were found for the trunk and upper arm postures during direct endoscopy: The necessity to hold a permanent contact of the endoscope with the eye and to grip the instrument with both hands (see the example shown in the left-hand photo of figure 1) results for taller persons in a lateral trunk inclination in the category '0° to 20°' for about 25% of the operation time, in comparison to less than 10% for the smaller persons. Right-upper-arm elevation is enhanced for the smaller persons; the percentage of time with upper-arm elevation above 60° amounts to more than 60% for the smaller persons in comparison to about 30% for the taller ones. Both, the long-term sideward inclination of the trunk and the elevation of the arm are significantly reduced when applying the monitor technique.

Conclusion of the posture data reveals that the head, trunk and arm positions are more disadvantageous during direct endoscopy than during monitor endoscopy. These findings match results from previous electromyographical studies in the operation theatre on shoulder and back muscles of surgeons (right and left m. trapezius, right m. deltoideus, left m. erector spinae) during the performance of urological operations [6,13]. Comparison of the myoelectrical activities for the different operation techniques demonstrates a significant decrease in particular for the shoulder muscles, when monitor endoscopy is applied instead of direct endoscopy. In the electromyographical studies also the occurrence of muscular fatigue was studied [14]. In case of direct endoscopy for about 80% of the operations muscular fatigue was verified, whereas for the application of monitor endoscopy the percentage of operations with fatigue was reduced to about 42% [15].

The performance of the fine-motoric work of transurethral tissue resection requires a stable positioning of the resectoscope. Steady holding of the instrument by means of the hands is only possible, if the upper body including the trunk, shoulders and arms represents a firm mechanical basis for the manipulations with the wire loop. The stabilisation of the positions of the upper body elements has to be performed by the muscles spanning the inter-segmental joints. Fixation of the links between adjacent segments can effectively be executed by co-contractions of the respective flexor and extensor muscles. Correspondingly, continuous muscular activation at a high level has to be expected. This assumption was confirmed in the aforementioned electromyographical studies [13] indicating very high activation levels for the trapezius and deltoideus muscles of up to about 60% of the maximum voluntary activation during direct endoscopy. In case of direct endoscopy, activation is enhanced, since the upper body has to be fixed in constrained postures with increased lateral and sagittal inclinations and long-term arm elevations up to shoulder height with the hands located in the height of the eyes, whereas during monitor applications the endoscope is held with almost hanging upper arms and the activation level of the trapezius and deltoideus is significantly lower [6]. Muscular strain was found to depend on the anthropometry of the surgeons: For surgeons with a body height above 180 cm higher muscular activities were observed in the shoulder region than for smaller persons [6]. The more disadvantageous trunk positions of the taller persons mentioned before and, in particular, the increased time fraction with lateral trunk inclinations are assumed as a possible reasen, even if the height of the operation table and the seat of the operating chair were commonly adjusted according to the individual anthropometry of the surgeon.

### Reduction of postural stress by ergonomic work design

With respect to the reduction of postural stress, a chair with back support was proven to be a successful tool. Back support was used for a considerable time of more than one third of the operation time and the reduction in the myoelectrical activity of the erector spinae found in the aforementioned studies may result at least partly from the use of the back support.

For the armrests, it is difficult to make a quantitative assessment regarding their benefit. Visual observation of the surgeons' postures gives the impression that the surgeons used the left-hand armrest very often. However, the time of use could not be quantified exactly in the off-line analysis of this study, since the left-hand side of the body was insufficiently displayed in the video recordings and the posture evaluation of this study was therefore confined to the right-hand body segments. The number of 3% of the operation time mentioned before for the use of the right-hand armrest is the only quantitative item determined in this regard. It is, however, not very meaningful without further interpretations, since the right hand is used to resect the tissue by means of the wire loop inserted in the resectoscope. During the execution of such fine hand and finger movements, the corresponding arm should not be posed on the armrest and, consequently, the right-hand armrest could be used for small periods of time, only. Even if the time for the use of the left-hand armrest was not determined quantitatively, its advantage seems to be obvious, since the left hand is used to stabilise the position of the resectoscope and the fixation of the instrument is effectively supported when the left elbow is placed on the armrest. The aforementioned reduction in the myoelectrical activity of the left trapezius after the redesign of the workplace including the use of an operation chair with armrests may also confirm this statement.

For the posture analyses performed in this study it seems to be important to consider the sensorimotor lateral preference of the subjects, i.e. of the eye, hand and foot. In particular the lateralisation of the eye in combination with the handedness may influence the postures of the upper body adopted during the operations [16]. It has to be assumed that, in particular during direct endoscopy, the head posture is affected by the eye preference, since the dominant eye is used to perform such monocular tasks. Also the lateralisation of the hand may interact with the trunk and shoulder posture since the resectoscope is normally held by the non-dominant hand whereas the dominant hand is used to manipulate the wire loop and to perform the fine-motoric work of tissue resection [17]. With respect to the sensorimotor lateral preference the studied group was consistent: All surgeons used the left hand to hold the instrument, the right hand to manipulate the wire loop and the right eye to contact the aperture of the endoscope when applying direct endoscopy. Therefore a possible interference of the results with different lateralisation of the subjects can be excluded in this study. In general, however, a relationship between the individual lateralisation status for eye, hand and foot and the preferred endoscopic operation technique (direct vs. monitor endoscopy) seems to be relevant according to a nation-wide survey on about 1350 urologists [16].

## Conclusion

From the ergonomic point of view, endoscopic transurethral prostate and bladder operations should preferably be performed using a video-assisted operation method (monitor endoscopy) instead of directly viewing at the operation area via the endoscope (direct endoscopy) for several reasons:

- During direct endoscopy, the percentage of the operation time and the duration of the periods with disadvantageous postures of the upper body are increased in comparison to monitor endoscopy. In particular, distinct lateral and sagittal inclinations of the trunk and head as well as torsion of the trunk are to be found. Furthermore, the upper arm and forearm are held in clearly elevated positions for longer periods and larger portions of time.

- In direct endoscopy, long-term trunk and head inclination as well as arm elevation are inevitable, since permanent contact between the eye and the endoscope is needed and, in consequence, the head and trunk have to track the endoscope movements during the total operation time.

- In monitor endocopy, the close coupling between the endoscope, the eye and the hands is solved and the application of the video system allows working in a more advantageous posture with hanging arms and an upright trunk and head.

- In direct endoscopy, muscular activity in the shoulder and back regions is enhanced and clear signs of muscular fatigue were established for the trapezius muscle in a former study. A considerable reduction of the myoelectrical activity was observed during monitor endoscopy. Furthermore, the percentage of operations with fatigue of the trapezius muscle was clearly reduced in comparison to the application of the direct-endoscopy methodology.

- The use of the monitor method allows the application of an operating chair equipped with back support and armrests and - in consequence - a reduction of postural stress, whereas in direct endoscopy the free movement of the trunk and arm is required and any trunk or back support is hindering.

## Competing interests

The authors declare that they have no competing interests.

## Authors' contributions

AL and MJ conceived and designed the study. JS suggested the study and provided the clinical expertise. AL prepared the manuscript. All authors have read and approved the manuscript.

## Supplementary Material

Additional file 1 **Table S1 - Encoding system for body segments' positions**. System for the categorisation of body segments' positions including the range of definition for the various movements and the used step-width and numbers of categories. Click here for file

Additional file 2 **Table S2 - Duration of body segments' positions**. Duration of the time periods the surgeons remained uninterruptedly in various posture categories of the head, trunk and right arm; average and range (in brackets) of the mean durations for the single operations (direct endoscopy: n = 10, monitor endoscopy: n = 9). Click here for file
