# Supplementary material for: Ergonomic assessment of the posture of surgeons performing endoscopic transurethral resections in urology
Source: J Occup Med Toxicol. 2009 Oct 19;4:26. doi: 10.1186/1745-6673-4-26 (PMC2770550; doi:10.1186/1745-6673-4-26)
Supplement: Additional file 1 — Table S1 - Encoding system for body segments' positions. System for the categorisation of body segments' positions including the range of definition for the various movements and the used step-width and numbers of categories. [file 1745-6673-4-26-S1.PDF]

digit	posture description	range	step-width	no. of steps
trunk positions				
1	sagittal trunk inclination	> 60° forward to > 20° backward	20°	7
2	lateral trunk inclination	> 40° rightward to > 40° leftward	20°	7
3	trunk torsion	> 20° rightward to > 20° leftward	20°	5
4	hollow back	yes / no	-	2
5	trunk support	yes / no	-	2
head positions				
6	sagittal head inclination	> 20° forward to > 10° backward	forward: 20°<br>backward: 10°	5
7	lateral head inclination	> 20° rightward to > 20° leftward	20°	5
8	head torsion	> 20° rightward to > 20° leftward	20°	5
9	head support	yes / no	-	5
shoulder and arm positions				
10	right-shoulder elevation	yes / no	-	2
11	right-arm ante- / retroversion	forward / no / backward	-	3
12	right-upper-arm elevation	around 0° to above shoulder height	20°	5
13	right-forearm elevation	> 20° downward to > 20° upward	20°	5
14	right-elbow flexion	around 0° to > 90°	45°	5
15	right arm support	yes / no	-	2
miscellaneous				
16	lower-body posture	sitting / standing	-	2
17	line of vision (monitor endoscopy)	monitor / endoscope / other	-	3
