# Supplementary material for: Ergonomic assessment of the posture of surgeons performing endoscopic transurethral resections in urology
Source: J Occup Med Toxicol. 2009 Oct 19;4:26. doi: 10.1186/1745-6673-4-26 (PMC2770550; doi:10.1186/1745-6673-4-26)
Supplement: Additional file 2 — Table S2 - Duration of body segments' positions. Duration of the time periods the surgeons remained uninterruptedly in various posture categories of the head, trunk and right arm; average and range (in brackets) of the mean durations for the single operations (direct endoscopy: n = 10, monitor endoscopy: n = 9). [file 1745-6673-4-26-S2.PDF]

**sagittal head inclination**						
**posture category**		< -10°	-10° to 0°	around 0°	0° to 20°	> 20°
			backward	← →	forward	
**duration**	direct endoscopy	0.5 (0.0 – 3.0) s	8.8 (2.3 – 13.5) s	3.5 (2.4 – 7.5) s	16.3 (6.7 – 32.1) s	2.7 (0.0 – 15.4) s
	monitor endoscopy	4.3 (0.0 – 28.5) s	14.1 (3.7 – 39.9) s	8.7 (1.5 – 32.7) s	4.3 (3.2 – 5.6) s	0.7 (0.0 – 3.0) s
**lateral head inclination**						
**posture category**		<-20°	-20° to 0°	around 0°	0° to 20°	>20°
			leftward	← →	rightward	
**duration**	direct endoscopy	0.1 (0.0 – 1.0) s	3.5 (2.4 – 8.7) s	9.2 (5.7 – 15.9) s	7.1 (4.2 – 10.4) s	8.1 (0.0 – 15.4) s
	monitor endoscopy	0.3 (0.0 – 3.0) s	5.2 (2.0 – 12.7) s	22.1 (12.1 – 61.1) s	4.4 (0.0 –10.0) s	0.7 (0.0 – 3.0) s
**sagittal trunk inclination**						
**posture category**		-20° to 0°	around 0°	0° to 20°	20° to 40°	40° to 60°
		backward	← →	forward		
**duration**	direct endoscopy	2.7 (1.0 – 8.0) s	6.7 (2.6 – 11.7) s	23.2 (15.2 – 33.4) s	12.6 (0.0 – 26.6) s	3.3 (0.0 – 10.0) s
	monitor endoscopy	2.7 (0.0 – 9.0) s	28.7 (10.5 – 75.4) s	9.4 (1.0 – 20.7) s	2.4 (0.0 – 7.0) s	0.4 (0.0 – 4.0) s
**lateral trunk inclination**						
**posture category**		-40° to -20°	-20° to 0°	around 0°	0° to 20°	20° to 40°
			leftward	← →	rightward	
**duration**	direct endoscopy	0.0 s	5.7 (2.8 – 13.5) s	15.3 (7.3 – 33.5) s	10.2 (4.4 – 14.4) s	7.2 (0.0 – 17.5) s
	monitor endoscopy	0.0 s	5.4 (1.3 – 12.3) s	24.1 (12.1 – 77.9) s	7.1 (0.0 – 10.9) s	0.0 s
**right-upper-arm elevation**						
**posture category**		around 0°	0° to 20°	20° to 60°	60° to 90°	> 90°
		→	upward			
**duration**	direct endoscopy	3.9 (0.0 – 15.0) s	5.9 (2.2 – 11.6) s	8.3 (4.6 – 12.7) s	14.2 (8.7 – 24.5) s	7.6 (2.0 – 11.7) s
	monitor endoscopy	4.4 (2.0 – 8.3) s	10.8 (2.4 – 25.7) s	9.6 (6.5 – 13.2) s	6.9 (1.9 – 18.0) s	0.8 (0.0 – 2.5) s
**right-forearm elevation**						
**posture category**		< -20°	-20° to 0°	around 0°	0° to 20°	> 20°
			downward	← →	upward	
**duration**	direct endoscopy	1.4 (0.0 – 3.7) s	4.0 (2.2 – 9.3) s	4.4 (0.0 – 14.9) s	4.1 (2.0 – 6.8) s	24.2 (11.6 – 58.7) s
	monitor endoscopy	0.7 (0.0 – 2.0) s	15.3 (0.0 – 60.0) s	4.8 (1.3 – 11.6) s	4.0 (1.9 – 6.1) s	7.9 (2.5 – 13.2) s
